# Paired Serum and Urine Concentrations of Biomarkers of Diethyl Phthalate, Methyl Paraben, and Triclosan in Rats

**DOI:** 10.1289/ehp.1409586

**Published:** 2015-06-05

**Authors:** Susan L. Teitelbaum, Qian Li, Luca Lambertini, Fiorella Belpoggi, Fabiana Manservisi, Laura Falcioni, Luciano Bua, Manori J. Silva, Xiaoyun Ye, Antonia M. Calafat, Jia Chen

**Affiliations:** 1 Department of Preventive Medicine, Icahn School of Medicine at Mount Sinai, New York, New York, USA; 2 Cesare Maltoni Cancer Research Center, Ramazzini Institute, Bologna, Italy; 3 National Center for Environmental Health, Centers for Disease Control and Prevention, Atlanta, Georgia, USA

## Abstract

**Background:**

Exposure to environmental chemicals, including phthalates and phenols such as parabens and triclosan, is ubiquitous within the U.S. general population.

**Objective:**

This proof-of-concept rodent study examined the relationship between oral doses of three widely used personal care product ingredients [diethyl phthalate (DEP), methyl paraben (MPB), and triclosan] and urine and serum concentrations of their respective biomarkers.

**Methods:**

Using female Sprague-Dawley rats, we carried out two rounds of experiments with oral gavage doses selected in accordance with no observed adverse effect levels (NOAELs) derived from previous studies: 1,735 (DEP), 1,050 (MPB), 50 (triclosan) mg/kg/day. Administered doses ranged from 0.005 to 173 mg/kg/day, 10–100,000 times below the NOAEL for each chemical. Controls for the MPB and triclosan experiments were animals treated with olive oil (vehicle) only; controls for the DEP serum experiments were animals treated with the lowest doses of MPB and triclosan. Doses were administered for 5 days with five rats in each treatment group. Urine and blood serum, collected on the last day of exposure, were analyzed for biomarkers. Relationships between oral dose and biomarker concentrations were assessed using linear regression.

**Results:**

Biomarkers were detected in all control urine samples at parts-per-billion levels, suggesting a low endemic environmental exposure to the three chemicals that could not be controlled even with all of the precautionary measures undertaken. Among the exposed animals, urinary concentrations of all three biomarkers were orders of magnitude higher than those in serum. A consistently positive linear relationship between oral dose and urinary concentration was observed (*R*^2^ > 0.80); this relationship was inconsistent in serum.

**Conclusions:**

Our study highlights the importance of carefully considering the oral dose used in animal experiments and provides useful information in selecting doses for future studies.

**Citation:**

Teitelbaum SL, Li Q, Lambertini L, Belpoggi F, Manservisi F, Falcioni L, Bua L, Silva MJ, Ye X, Calafat AM, Chen J. 2016. Paired serum and urine concentrations of biomarkers of diethyl phthalate, methyl paraben, and triclosan in rats. Environ Health Perspect 124:39–45; http://dx.doi.org/10.1289/ehp.1409586

## Introduction

Exposure to environmental chemicals, including phthalates and phenols such as parabens and triclosan, is ubiquitous within the U.S. general population [Centers for Disease Control and Prevention (CDC) 2014]. There is growing interest in the possible adverse human health outcomes associated with exposure to these chemicals (Diamanti-Kandarakis et al. 2009).

Phthalates are used in a wide range of consumer goods (CDC 2014). Low molecular weight phthalates [e.g., diethyl phthalate (DEP)] are often found in personal care products (e.g., fragrances, shampoo, cosmetics). In rats, DEP is hydrolyzed to its monoester metabolite, monoethyl phthalate (MEP) (Albro and Moore 1974); DEP metabolism is assumed to be similar in humans (ATSDR 1995). Elimination half-lives of DEP and MEP in mammals have not been experimentally defined but are believed to be a few hours (Calafat and McKee 2006). Therefore, MEP has been used as a biomarker of recent exposure to DEP. Several health effects have been associated with elevated urinary concentrations of MEP, including adverse male reproductive outcomes (Duty et al. 2003; Hauser et al. 2007; Jönsson et al. 2005; Swan et al. 2005), altered neonatal behavior and neurobehavioral development (Engel et al. 2009, 2010; Wolff et al. 2008), and increased breast cancer risk (López-Carrillo et al. 2010).

Parabens are commonly used as preservatives in personal care products, cosmetics, pharmaceuticals, and even in the processing of foods and beverages (Golden et al. 2005). Most Americans are exposed to various parabens, including methyl paraben (MPB) (CDC 2014). Because of their potential estrogenic activity, parabens have been suggested to play a role in breast cancer, albeit orders of magnitude lower than that of endogenous estrogens (Darbre and Harvey 2008; Golden et al. 2005); however, strong epidemiologic evidence is lacking (McGrath 2003; Mirick et al. 2002). Parabens are hydrolyzed to *p*-hydroxybenzoic acid, which can be conjugated and excreted in urine. *p*-Hydroxybenzoic acid and its conjugates are nonspecific metabolites of all parabens (Ye et al. 2006). However, the concentrations of total (free plus conjugated) urinary species of the parent parabens are considered valid biomarkers of paraben exposure in humans (Ye et al. 2006) and have been used as measures of environmental exposure to these chemicals in epidemiologic studies (Mervish et al. 2014; Wolff et al. 2010, 2014).

Triclosan is a commonly used antimicrobial in personal care and household products ranging from toothpaste, deodorant, and hand soap to cutting boards and textiles [U.S. Department of Health and Human Services (DHHS) 2015]. Public interest has been steadily increasing in the ubiquitous sources of exposure to this chemical. The hormonal activity of triclosan has not been clearly established owing to conflicting results from different investigations, including evidence of weak estrogenic (Chen et al. 2007; Svobodová et al. 2009) and androgenic activity (Chen et al. 2007), estrogen receptor antagonism (Ahn et al. 2008), and anti-androgenic properties (Chen et al. 2007). The excretion half-life of triclosan has been estimated as 11 hr for urine and 21 hr for plasma (Sandborgh-Englund et al. 2006). When excreted in urine, triclosan is mainly in its conjugated form, whereas the percentage of free triclosan is higher in plasma (Sandborgh-Englund et al. 2006). Urinary concentrations of triclosan (conjugated plus free species) can be used as a biomarker of exposure (Calafat et al. 2008).

Given the variability in the bioactivities of these chemicals, understanding the dose–response relationship is fundamental and essential for studying their potential biologic or health effects. Furthermore, the dose–response relationship may depart from linearity at low doses (Vandenberg et al. 2012), making dose extrapolation difficult and potentially unreliable. Thus, to evaluate risks or investigate the biological effects of environmental chemicals, it is critical to employ doses in animal experiments that are comparable to the human experience. However, the dose ranges used for DEP, MPB, and triclosan in animal studies have been wide and often orders of magnitude higher than humans are likely to encounter (Shiraishi et al. 2006; Stoker et al. 2010; Vo et al. 2010). On the basis of increasing evidence suggesting low-dose health effects of these chemicals, there is an urgent need for studies that utilize doses in the range of typical human exposure (Birnbaum 2012; Casals-Casas and Desvergne 2011).

The objective of this study was to investigate the relationship between oral doses of three widely used personal care product ingredients (DEP, MPB, and triclosan) in rats and the resulting urinary and serum biomarker concentrations. We used a traditional rat model, which is commonly employed in toxicology and risk assessment research. The rat has been used extensively for research in developmental and reproductive physiology and endocrinology and has been more thoroughly characterized in these research fields than other species, likewise for identifying likely human carcinogens (Gray et al. 2004; Maltoni et al. 1999). Although these experiments are part of a larger study examining personal care product ingredients and breast cancer risk, the results of our study will provide a foundation for future rodent-based health risk assessment studies for human exposure to these chemicals.

## Methods

*Materials and standards.* Diethyl phthalate (CAS 84-66-2, lot STBB0862V, 99% purity), methyl paraben (CAS 99-76-3, lot BCBG0852V, 99% purity), and triclosan (CAS 3380-34-5, lot 1412854V, 97% purity) were supplied by Sigma Aldrich (Milan, Italy). Olive oil (lot 111275; Montalbano Agricola Alimentare Toscana, Florence, Italy) was used as vehicle to prepare all dosing solutions. During the experiment, each compound was stored in the dark at room temperature (20°C). The solutions to be used for the entire experiment (5 days) were prepared on the first day of treatment and were continuously stirred throughout the study; the stability of the solutions was confirmed by gas chromatography–mass spectrometry (GC/MS) (Neotron Laboratory, Modena, Italy).

*Experimental animals.* All animal study procedures were performed in accordance with the guidelines of the Institutional Animal Care and Use Committee (IACUC), following the principles of the Good Laboratory Practices and Standard Operating Procedure of the Ramazzini Institute (RI) facility. The protocol was also approved by the Mount Sinai IACUC. The animals were treated humanely and with regard for alleviation of suffering. This study was designed as a proof-of-concept experiment to determine oral doses to be used in a larger investigation focusing on the potential effects of these chemicals on mammary tissue gene expression; therefore, only female Sprague-Dawley (S-D) rats were used for this study. This strain belongs to the colony that has been used for over 40 years in the laboratory of the Cesare Maltoni Cancer Research Centre of the RI facility. The animals were randomly distributed into 10 groups in order to minimize the number of animals from each litter in the same group. Rats were identified by ear punch in accordance with the Jackson Laboratory system. Throughout the treatment period before urine and blood collection, animals were housed in standard polycarbonate cages (41 cm × 25 cm × 15 cm) with stainless steel wire tops and a shallow layer of white wood shavings for bedding; two or three rats were housed per cage. Cages were identified by cards indicating the experiment, the group, and the experimental number/pedigree number of each animal. During each experiment, all animals were kept in a single room at 23°C ± 3°C and 40–60% relative humidity. Lighting was natural or artificial to maintain a 12 hr light/dark cycle. Before treatment was started, animals were weighed and dosed based on the average weight of the experimental group (milligrams per kilogram body weight). Rat feed (Laboratorio Dr Piccioni, Milan, Italy) and tap water were provided *ad libitum*. Each lot of feed and tap water was periodically analyzed for biologicals (bacteria) and chemicals (mycotoxins, pesticides, arsenic, lead, mercury, selenium) but not for DEP, triclosan, or MPB. The experimental design is shown in Table 1. To reduce the possibility of DEP, triclosan, or MPB contamination, all cages, containers, and syringes used during the experiments were washed without detergent; instead, these items were cleaned by exposure to hot water for an extended period of time.

**Table 1:** Administered doses and available biological samples.

Compounds	Administered dose^*a*^			Samples^*b*^	
	Reference	mg/kg/day	Rats (*n*)	Urine	Serum
Experiment 1
Diethyl phthalate	NOAEL/10	173.5	5	Yes	No
	NOAEL/100	17.35	5	Yes	No
	NOAEL/200	8.68	5	Yes	No
Methyl paraben	NOAEL/10	105	5	Yes	No
	NOAEL/100	10.5	5	Yes	No
	NOAEL/200	5.25	5	Yes	No
Triclosan	NOAEL/10	5	5	Yes	No
	NOAEL/100	0.5	5	Yes	No
	NOAEL/200	0.25	5	Yes	No
Control	Olive oil	—	5	Yes	No
Experiment 2
Diethyl phthalate	NOAEL/200	8.675	5	Yes	Yes
	NOAEL/10,000	0.1735	5	Yes	Yes
	NOAEL/100,000	0.01735	5	Yes	Yes
Methyl paraben	NOAEL/200	5.25	5	Yes	Yes
	NOAEL/10,000	0.105	5	Yes	Yes
	NOAEL/100,000	0.0105	5	Yes	Yes
Triclosan	NOAEL/200	0.25	5	Yes	Yes
	NOAEL/1,000	0.05	5	Yes	Yes
	NOAEL/10,000	0.005	5	Yes	Yes
Control	Olive oil	—	5	Yes	Yes
NOAEL, no observed adverse effect level. ^***a***^NOAEL (mg/kg/day) for diethyl phthalate = 1,735; methyl paraben = 1,050; triclosan = 50. ^***b***^Only pooled urine samples available in experiment 1; individual urine and serum samples available in experiment 2.					

*Chemical treatment.* Two rounds of experiments (Table 1) were performed to identify the oral gavage–administered dose of each chemical that resulted in urinary concentrations of the corresponding biomarker within the ranges reported in the U.S. National Health and Nutrition Examination Survey (NHANES) (CDC 2014). For each round, three doses were selected for each chemical according to no observed adverse effect levels (NOAELs) that were defined based on previous studies as 1,735 mg/kg/day for DEP (Brown et al. 1978; Moody and Reddy 1978; Oishi and Hiraga 1980), 1,050 mg/kg/day for MPB (U.S. EPA 2005), and 50 mg/kg/day for triclosan (Goldsmith and Craig 1983; Rodriguez and Sanchez 2010).

Female S-D rats were treated by oral gavage daily for 5 days. Each treatment group consisted of 5 rats, including a control group with rats treated with olive oil only. A total of 50 rats in 10 experimental groups (three chemicals × three doses + one control) were involved in each experimental round. For both experimental rounds, the dosing day was designated as day 0, and urine and serum samples were collected on day 6. The first experiment was carried out with rats at 16 weeks of age; the selected testing doses were NOAEL/10, NOAEL/100, and NOAEL/200 for each of the three target chemicals. The second experiment was carried out with rats at 27 weeks of age; the testing doses were NOAEL/200, NOAEL/10,000, and NOAEL/100,000 for DEP and MPB, and NOAEL/200, NOAEL/1,000, and NOAEL/10,000 for triclosan.

To minimize external contamination, the olive oil and chemicals were stored in glass containers and administered using 5-mL glass syringes. DEP, triclosan, and MPB were not detected in the olive oil used as vehicle using GC/MS at Neotron Laboratory, Modena, Italy (http://www.neotron.it). Biological samples were collected in polypropylene vials. At the end of the experiment and after the urine collection (day 6), each rat was sacrificed by CO_2_ inhalation.

*Urine collection.* The morning after completing treatment (day 5), rats were moved from their experimental cages to metabolic cages (TECNIPLAST S.p.A., Italy), where each rat was individually housed for 24 hr without food but with water *ad libitum*. Metabolic cages are specifically designed to prevent fecal contamination of urine. Twenty-four-hour urine was collected and placed in pre-labeled polypropylene vials. Furthermore, a single dose-group urine pool composed of 200 μL of urine from each animal in the group was created and stored in a separate vial. Urine vials were placed in cryoboxes and frozen at –20°C, then shipped on dry ice to the National Center for Environmental Health (NCEH) laboratory at the Centers for Disease Control and Prevention (CDC). Upon arrival, the samples were stored at –70°C until analysis. Only pooled urine samples from the first experiment were available for analysis; individual and pooled urine samples were available for the second experiment.

*Serum collection.* Serum samples were collected only for the second experiment. After sacrifice, whole blood (5 mL) was collected from the inferior vena cava using a 5-mL glass syringe, placed in a tube without anticlotting agents, and left at room temperature for 30 min. The clot was removed by centrifugation at 2,500 × *g* for 10 min. The serum (supernatant) was transferred into a clean polypropylene vial using a glass Pasteur pipette. The serum samples were placed in a cryobox and frozen at –20°C, then shipped on dry ice to the CDC’s NCEH laboratory. Upon arrival, the samples were stored at –70°C until analysis.

*Laboratory analysis.* Urine and serum samples were analyzed at the CDC’s NCEH laboratories for MEP, the common biomarker of DEP, triclosan, and MPB. MEP, the specific monoester metabolite of DEP, is measured as a biomarker of exposure to DEP because measurement of the parent compound poses a number of challenges (Koch and Calafat 2009). Phthalate diesters are ubiquitous in the environment, can be detected in the laboratory setting, and are quickly metabolized into their hydrolytic monoester (Koch and Calafat 2009). Therefore, MEP is the preferred biomarker because it is not as prone to contamination (Koch and Calafat 2009). Analytical methods for these three biomarkers (MEP, MPB, and triclosan) have been published (Silva et al. 2007a; Ye et al. 2005, 2008). Conjugated species of the biomarkers were enzymatically hydrolyzed, pre-concentrated by on-line solid phase extraction, and separated from other matrix components by high performance liquid chromatography. Quantitation was achieved by isotope dilution tandem mass spectrometry. Limits of detection (LOD), calculated as three times the standard deviation as the concentration approaches zero (Taylor 1987), for MEP, MPB, and triclosan were 0.6, 1.0, 2.3 ng/mL in urine, and 0.6, 0.1, 1.1 ng/mL in serum, respectively. Standards, quality control samples, and reagent blanks were included in each analytical batch along with the experimental samples. Quality control samples were evaluated in accordance with standard statistical probability rules (Caudill et al. 2008).

*Statistical methods.* For the second experiment, where individual urine and serum samples were available, we examined the associations between oral dose and individual urinary/serum biomarker concentration using linear regression models. Values below the LOD were imputed as LOD divided by the square root of two. We also examined the relationship between urinary and serum biomarker concentrations using Spearman correlation coefficients. Serum MEP concentrations in samples from controls treated with olive oil (mean, 136.2 ± 37.8 ng/mL) were greater than serum concentrations measured in samples from animals with the lowest DEP dose (mean, 15.8 ± 8.9 ng/mL), indicating a potential contamination problem. Investigation of both the animal experiment protocol and the analytic laboratory protocol did not reveal any obvious reasons for these results. To avoid sacrificing additional animals, a decision was made to use the MEP concentration in serum from the 10 rats in the lowest dose categories of the triclosan and MPB groups as the control concentrations in the regression analyses for the association between oral DEP and serum MEP. Statistical analyses were performed using SAS 9.3 for Windows (SAS Institute Inc., Cary, NC, USA).

Biomarker concentrations measured in the rats’ urine were compared with the 2009–2010 National Health and Nutrition Examination Survey urine concentration median and 95th percentile (ng/mL) for females. These concentrations were 59.6 and 988, respectively, for MEP; 106 and 1,230, respectively, for MPB; and 10.5 and 488, resepctively, for triclosan (CDC 2014).

## Results

**Figure 1:**
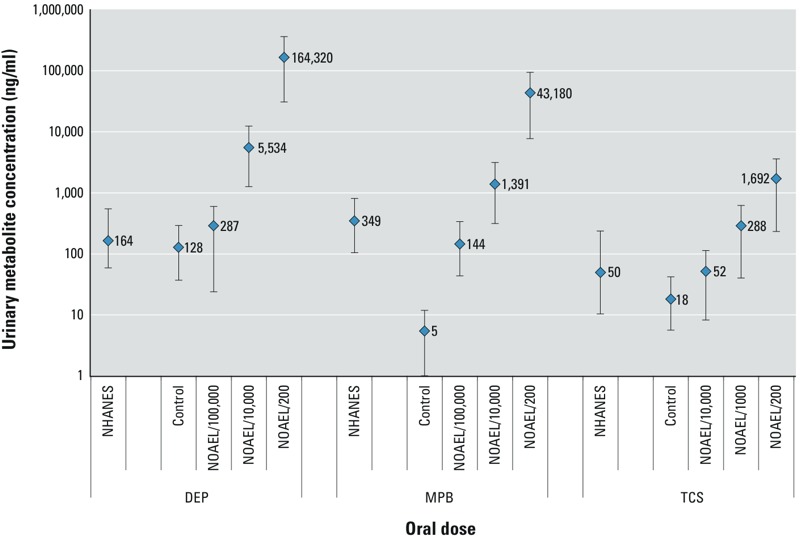
Urinary metabolite concentrations of monoethyl phthalate, methyl paraben, and triclosan by oral dose (*n *= 5 rats per dose group) and in NHANES (2009–2010). Abbreviations: DEP, diethyl phthalate; MPB, methyl paraben; NHANES, National Health and Nutrition Examination Survey; NOAEL, no observed adverse effects level; TCS, triclosan. For oral doses of DEP, monoethyl phthalate (MEP) is measured as the biomarker and presented as the urinary metabolite concentration. Data represent the 50th, 75th, and 90th percentiles of urine concentrations (in ng/mL) for the U.S. female population (> 6 years old) from NHANES (2009–2010). All other data represent the mean ± SD for urine concentrations in experimental animals.

*Associations between urinary metabolite concentration and oral dose.* In the first experiment, only a single pooled urine sample from each experimental group (five rats) was analyzed for MEP, MPB, or triclosan. The results are presented in Table 2. The underlying goal of these experiments was to find oral doses of the three chemicals that resulted in urinary biomarker concentrations in the range reported for the U.S. female population. All dose groups in the first experiment had concentrations that greatly exceeded the 95th percentile (100 times) and the geometric mean (1,000 times) as reported in NHANES (CDC 2014). Therefore, we conducted a second experiment using much lower doses: NOAEL/200, NOAEL/10,000, and NOAEL/100,000 for DEP and MPB, and NOAEL/200, NOAEL/1,000, and NOAEL/10,000 for triclosan. In contrast to the first set of experiments, individual urine samples from the five rats in each treatment group were analyzed. Figure 1 presents the mean (± standard deviation) for urinary concentrations of MEP, MPB, and triclosan for each dose group. For all three chemicals, the mean urinary concentrations for the lower oral doses were in the range of urinary concentrations reported for the U.S. female general population (CDC 2014). As shown in Table 3, the urinary concentrations demonstrated a strong linear relationship with the oral doses for each of the three chemicals. Moreover, all *R*^2^ values were greater than 0.80 (*p* < 0.001).

**Table 2:** Urinary and serum biomarker concentrations by administered oral doses of diethyl phthalate, methyl paraben, and triclosan.

Biomarker	Experiment 1			Experiment 2				
	Oral dose NOAEL/X	Oral dose (mg/kg/day)	Urine concentration (ng/mL), pooled sample^*a*^	Oral dose NOAEL/X	Oral dose (mg/kg/day)	Urine concentration (ng/mL) Mean ± SD^*b*^	Serum *n* > LOD/ total samples	Serum concentration (ng/mL) Mean ± SD
Monoethyl phthalate (MEP)^*c*^
	10	173.5	2.02 × 10^6^	200	8.68	1.64 × 10^5^ ± 6.97 × 10^4^	5/5	19.12 ± 7.56
	100	17.35	2.43 × 10^5^	10,000	0.174	5.53 × 10^3^ ± 2.87 × 10^3^	5/5	21.66 ± 9.21
	200	8.68	2.28 × 10^5^	100,000	0.0174	2.87 × 10^2^ ± 5.31 × 10^1^	5/5	15.76 ± 8.89
		0^*d*^	3.22 × 10^3^		0^*e*^	1.28 × 10^2^ ± 8.24 × 10^1^	10/10^*f*^	12.26 ± 2.37
Methyl paraben (MPB)
	10	105	5.00 × 10^5^	200	5.25	4.32 × 10^4^ ± 1.74 × 10^4^	5/5	1.64 ± 0.48
	100	10.5	9.02 × 10^4^	10,000	0.105	1.39 × 10^3^ ± 7.09 × 10^2^	1/5	0.4^*g*^
	200	5.25	7.70 × 10^4^	100,000	0.0105	1.44 × 10^2^ ± 9.72 × 10^1^	1/5	0.4^*g*^
		0^*d*^	3.85 × 10^0^		0^*e*^	5.46 × 10^0^ ± 2.27 × 10^0^	5/5	0.96 ± 0.44
Triclosan (TCS)
	10	5	7.91 × 10^3^	200	0.25	1.70 × 10^3^ ± 5.19 × 10^2^	5/5	186 ± 40.69
	100	0.5	1.28 × 10^3^	1,000	0.05	2.88 × 10^2^ ± 9.02 × 10^1^	5/5	46.88 ± 20.76
	200	0.25	5.94 × 10^2^	10,000	0.005	5.22 × 10^1^ ± 1.86 × 10^1^	5/5	4.72 ± 2.20
		0^*d*^	5.4 × 10^0^		0^*e*^	1.81 × 10^1^ ± 1.27 × 10^1^	0/5	—
Abbreviations: LOD, Limit of detection; NOAEL, no observed adverse effect level. ^***a***^Biomarker concentration measured in a single sample from a urine pool from five individual rats for each dose group. ^***b***^Mean of biomarker concentrations measured in five individual urine samples for each dose group. All biomarkers measured in urine samples were detectable (> LOD); LOD (ng/mL) in urine for MEP, MPB, and TCS were 0.6, 1.0, and 2.3, respectively. For comparison, the 2009–2010 National Health and Nutrition Examination Survey urine concentration median and 95th percentile (ng/mL) for females were 59.6 and 988, respectively, for MEP; 106 and 1,230, respectively, for MPB; and 10.5 and 488, respectively, for triclosan (CDC 2014). ^***c***^Monoethyl phthalate was used as the biomarker of exposure to diethyl phthalate. ^***d***^One control group of 5 rats was used for experiment 1; the pooled urine sample was analyzed for all three biomarkers (MEP, MPB, and triclosan). ^***e***^One control group of 5 rats was used for experiment 2; the 5 individual urine samples were analyzed for all three biomarkers (MEP, MPB, and triclosan). ^***f***^MEP serum controls: MEP concentrations measured in serum from 5 MPB and 5 TCS animals treated with the lowest oral dose (MPB: NOAEL/100,000; TCS: NOAEL/10,000); see “Methods” for details. ^***g***^Concentration measured in the single serum sample among dose group with measurement > LOD; LOD (ng/mL) in serum for MEP, MPB, and triclosan were 0.6, 0.1, and 1.1, respectively.								

**Table 3:** Relationship of urinary and serum biomarker metabolites with orally administered dose of diethyl phthalate, methyl paraben, and triclosan.

Exposure (oral dose)	Outcome (metabolite)	*R*^2^	
		Urine	Serum
DEP	MEP	0.84	0.04
MPB	MPB	0.85	0.57
TCS	TCS	0.88	0.88
Abbreviations: DEP, diethyl phthalate; MEP, monoethyl phthalate; MPB, methyl paraben; TCS, triclosan.			

*Associations between metabolite serum concentration and oral dose.* In the second experiment, we examined the relationship between serum concentration and oral dose of the three chemicals. Although MEP was detected in all serum samples, MPB was detected in 60% of the serum samples (Table 2). Triclosan was detected in the serum of all dosed animals, whereas all control animals had undetectable serum triclosan concentrations. In general, the concentrations of MEP, MPB, and triclosan in urine were considerably higher than those in serum: ~ 10^5^ times higher for MEP, ~ 10^4^ times higher for MPB, and ~ 10 times higher for triclosan. Spearman correlation coefficients between urine and serum concentrations were 0.44 (MEP), 0.23 (MPB), and 0.98 (triclosan). As shown in Table 3, a relationship between oral dose and biological concentration of MEP was present in urine only (*R*^2^ = 0.84 in urine and 0.04 in serum). For MPB, this relationship existed in both urine and serum but was stronger in urine (*R*^2^ = 0.85 in urine and 0.57 in serum). The decreased strength of the relationship in serum may be because of the higher detection frequency in the controls than in the two lower oral doses. For triclosan, the relationship was equally strong in both fluids (*R*^2^ = 0.88 in urine and 0.88 in serum).

## Discussion

We carried out a systematic dosing study in a rodent model and identified oral doses of three commonly used personal care product ingredients that resulted in comparable urinary biomarker concentrations to those observed in the U.S. female population. The results of this study may provide important information for future risk assessments of these chemicals that can be reliably translated to human populations.

The true daily intake of DEP, MPB, and triclosan by humans is unknown; however, several efforts have been made to estimate intake, and the oral doses used in experiment 2 compare well. For example, the NOAEL/100,000 experimental oral dose for both DEP and MPB (17.4 and 10.5 μg/kg/day, respectively) were only one order of magnitude higher than the estimated median (95th percentile in micrograms per kilogram per day) daily intake estimates of 1.7 (25) for DEP in the general Canadian female population ages 20–39 (Saravanabhavan et al. 2014) and 0.13 (0.36) for MPB in the general Chinese adult female population (Liao et al. 2013). The lowest oral dose of triclosan (NOAEL/10,000 = 0.5 μg/kg/day) was within the range of the estimated median (90th percentile in micrograms per kilogram per day) daily intake among an adult Belgian study population [0.017 (0.565)] (Geens et al. 2015). Estimation of daily intake depends on knowledge of the toxicokinetics, including administration, distribution, metabolism, and excretion (Søeborg et al. 2014); not all of this information was necessarily available to the cited authors for performing these calculations. However, the doses used in the second round of experiments do fall within the range of estimates calculated from adult human populations and provide support for the use of these oral doses in future translational animal experiments.

When conducting exposure studies with low doses, contamination can be a concern, especially when the chemicals being studied are ubiquitous in the environment. Careful protocols were implemented in the animal laboratory to minimize all potential sources of contamination as much as possible. These precautions included ensuring that the wearing of perfume and other scented products was avoided by staff, testing the olive oil (vehicle) for the presence of the three chemicals, using glass storage containers and glass syringes for all chemicals and solutions, and employing cleaning procedures that used only hot water without detergent. Fecal contamination of the urine could have occurred, although this source of contamination was highly unlikely owing to the design of the metabolic cages used to collect the urine samples. Similar strict protocols were also implemented in the analytic laboratory to avoid external contamination with the target biomarkers (Ye et al. 2013).

The detection of trace concentrations (i.e., parts per billion) of the biomarkers in all of the control urine samples suggests low endemic environmental exposure of the three chemicals that could not be controlled even with the precautionary measures undertaken. However, the magnitude of the possible contamination was far below the measured concentrations in the majority of urine samples from rats that received the experimental doses. The measured urine and serum concentrations likely resulted from a combination of the administered dose and the low endemic environmental dose. Future studies to repeat or extend the approaches presented herein would benefit from the use of stable-isotope-labeled chemicals in the dosing solution so that the biomarker measurements could be confidently attributed to the administered chemical exposure. To further investigate the unexpected detection of MEP in the serum from the 5 control rats (see Supplemental Material, Table S1, for individual animal results), samples from animals in the lowest dose groups of the other two chemicals (MPB and triclosan) were analyzed for MEP. The assumption was that these rats would be suitable controls if no MEP contamination had occurred. Trace MEP concentrations were similar among these 10 rats and were slightly lower than the serum concentrations of the group of rats exposed to the lowest DEP dose.

This study was designed to be a dose-calibration investigation. This type of study is typically smaller than a main experiment and therefore can provide only limited information about the sources and magnitude of variation of responses. The sample size was in line with the number of animals per dose group suggested by the Organisation for Economic Co-operation and Development (OECD) guidelines (OECD 2010) for toxicokinetic studies. The variability observed among the identically dosed rats is expected owing to inter-individual differences in absorption, distribution, metabolism, and excretion of the target analytes, in addition to differences in the water intake of the animals during the experiment; the same variability would be expected in humans. Although the same oral dose (NOAEL/200) was administered in experiments 1 and 2, the differences in urinary concentrations between the two experiments likely reflect the normal variability within the animals, that the experiments were performed at different times, and that the animals were of different ages at the start of each experiment. Thus, the two experiments should be considered individually. Variability in the concentrations of these metabolites among dosed animals has been observed in S-D rats dosed with dibutyl phthalate and di(2-ethylhexyl) phthalate (Calafat et al. 2006; Silva et al. 2007b), other phthalates (Silva et al. 2011), and other non-persistent chemicals, specifically phthalate alternatives (Silva et al. 2012).

Gavage is preferred over other routes of exposure to environmental chemicals when very low doses are used (Vandenberg et al. 2014). It is difficult to ascertain the true intake when chemicals are mixed into food or drinking water *ad libitum*. Although gavage does not perfectly represent a model of human dietary exposure, this route has been employed in numerous studies assessing potential carcinogenic hazards (Perera et al. 1989).

Animal models are useful tools for risk assessment of toxic chemicals and for identification of their potential physiologic consequences. Given the uncertainty of dose extrapolation, it is ideal to perform risk assessment using exposure conditions that mimic human experience. We chose the S-D rat as our model system because it has been shown to be one of the most physiologically relevant and genetically defined animal models for studying human sporadic breast cancer (Maltoni et al. 1996, 1997). The S-D rat model from the RI colony could be considered a human-equivalent model, particularly for breast lesions (non-neoplastic, pre-neoplastic, and neoplastic), that will allow us to translate rodent data to humans in future research (Teitelbaum et al. 2015). Furthermore, endocrine effects, which may occur even at the lowest doses, are difficult to detect without a highly sensitive experimental model. S-D rats are extremely sensitive and are recommended by the Endocrine Disruptor Screening Program of the U.S. Enviromental Protection Agency (EPA), which considers these animals particularly appropriate and relevant for identifying, extrapolating, and predicting likely effects in humans (U.S. EPA 2009). In a review of the literature, we found that the range of oral doses for DEP, MPB, and triclosan used in animal experiments was wide and, in almost all cases, much greater than the doses used in our exposure study (see Supplemental Material, Table S2, for a list of these studies). For example, in an investigation of estrogen-dependent responses to triclosan exposure in rats, triclosan was administered by oral gavage from postnatal day (PND) 19 to 21, and the oral dose ranged from 1.18 to 300 mg/kg/day (Stoker et al. 2010). In a study investigating MPB exposure and estrogenic effects, MPB was administered by oral gavage from PND 21 to 40 in oral doses ranging from 62.5 to 1,000 mg/kg/day (Vo et al. 2010). In another investigation of DEP exposure and endocrine-mediated properties, the chemical was administered by oral gavage for 28 days in doses ranging from 40 to 1,000 mg/kg/day (Shiraishi et al. 2006). In most cases, the lowest doses far exceeded the highest doses (0.25 mg/kg/day for triclosan, 5.25 mg/kg/day for MPB, 8.675 mg/kg/day for DEP) used in the present study. Importantly, only the lowest doses in our study, which were 500–5,000 times lower than the highest dose, resulted in biological levels that were comparable to those reported by NHANES. Therefore, most published studies employ doses that are not likely to be representative of exposures experienced by the U.S. population and are likely to be orders of magnitude higher.

The NOAEL, which can be determined either by experiment or through observation, denotes the highest level of exposure at which there is no biologically or statistically significant increase in the frequency or severity of any adverse effects in an organism when compared with a control (IPCS 1990). In the present study, it was necessary to administer oral doses that were many orders of magnitude lower than the NOAEL to observe urinary metabolite concentrations that were within the ranges reported for the U.S. population in NHANES 2009–2010 (CDC 2014). As noted above, the potential low endemic environmental exposure must be considered when evaluating the oral administered dose, highlighting the importance of identifying doses that are a realistic representation of human exposure to these chemicals to aid in the design of future animal experiments.

The results of this study indicate that oral exposure to three commonly used environmental chemicals, that is, DEP, MPB, and triclosan, has a strong linear relationship with the urinary concentrations of their corresponding biomarkers, but this relationship is not as uniformly strong in serum. The relatively weak association between administered oral gavage dose of DEP and MPB and the serum concentrations of their biomarkers exemplifies why there is growing concern about the use of the proper biologic matrix for exposure assessment (Calafat et al. 2013). The timing of urine and serum collection was the same, that is, 24 hr after completing treatment; however, urine was collected over a 24-hr period, and blood was collected as a spot sample. The half-lives of the investigated chemicals are relatively short, such that the interval between exposure and biomarker measurement may influence the findings if half-lives differ according to biologic matrix. Moreover, in general, the concentrations of MEP, MPB, and triclosan were much lower in serum than in urine, which is in accord with results obtained in human studies (Frederiksen et al. 2010). Taken together, the positive linear association of oral dose with urinary biomarkers, the higher percentages of urinary biomarkers detected compared with serum biomarkers, and the relatively low concentration of biomarkers in serum all support the use of urine as the appropriate biologic matrix for assessing exposure to these non-persistent chemicals.

## Conclusion

In this study, we identified a range of oral doses of three common environmental chemicals that result in urinary biomarker concentrations in a rat model that are consistent with the biomarker concentrations measured in the U.S. female population. Although endemic environmental exposure to the parent chemicals may have contributed to the biomarker concentrations measured in the rodents’ urine, the results of this study highlight the importance of carefully considering the oral dose used in animal experiments and provide useful information for selecting doses of DEP, MPB, and triclosan in future studies that evaluate their biological effects in experimental settings.

Editor’s Note: Information that was provided in the Advance Publication regarding NOAEL values defined for DEP and triclosan has been revised to indicate the individual studies on which the NOAELs were based, including Brown et al. (1978), Moody and Reddy (1978), and Oishi and Hiraga (1980) for DEP; and Goldsmith and Craig (1983) and Rodriguez and Sanchez (2010) for triclosan.

## Supplemental Material

(237 KB) PDF
