# Supplementary material for: Paired Serum and Urine Concentrations of Biomarkers of Diethyl Phthalate, Methyl Paraben, and Triclosan in Rats
Source: Environ Health Perspect. 2015 Jun 5;124(1):39–45. doi: 10.1289/ehp.1409586 (PMC4710607; doi:10.1289/ehp.1409586)
Supplement: (237 KB) PDF [file ehp.1409586.s001.acco.pdf]

Environ Health Perspect
DOI: 10.1289/ehp.1409586

Note to Readers: *EHP* strives to ensure that all journal content is accessible to all readers. However, some figures and Supplemental Material published in *EHP* articles may not conform to 508 standards due to the complexity of the information being presented. If you need assistance accessing journal content, please contact ehp508@niehs.nih.gov. Our staff will work with you to assess and meet your accessibility needs within 3 working days.

Supplemental Material

Paired Serum and Urine Concentrations of Biomarkers of Diethyl Phthalate, Methyl Paraben, and Triclosan in Rats

Susan L. Teitelbaum, Qian Li, Luca Lambertini, Fiorella Belpoggi, Fabiana Manservisi, Laura Falcioni, Luciano Bua, Manori J. Silva, Xiaoyun Ye, Antonia M. Calafat, and Jia Chen

Table of Contents

Table S1. Individual animal serum biomarker concentrations (ng/ml).

Table S2. Literature for animal studies on triclosan, diethyl phthalate and methyl paraben.

References

Table S1. Individual animal serum biomarker concentrations (ng/ml).

Experimental Groups - Administered chemical	Serum Concentration (ng/ml) Mono-ethyl phthalate	Methyl paraben	Triclosan
**Olive oil alone**			
Sample I	181	0.7	<LOD
Sample II	155	1.7	<LOD
Sample III	79	0.6	<LOD
Sample IV	139	1	<LOD
Sample V	127	0.8	<LOD
**Methyl paraben**			
NOAEL/100,000, 0.0105 mg/Kg/day			
Sample I	9.1	2.4	<LOD
Sample II	11.1	1.8	<LOD
Sample III	9.8	1.3	<LOD
Sample IV	11.7	1.2	<LOD
Sample V	11.3	1.5	<LOD
**Triclosan**			
NOAEL/10,000, 0.0005 mg/Kg/day			
Sample I	16.2	<LOD	190
Sample II	14.4	<LOD	206
Sample III	15.5	<LOD	152
Sample IV	12.3	<LOD	141
Sample V	11.2	<LOD	241
**Diethyl phthalate**			
NOAEL/10,000, 0.01735 mg/Kg/day			
Sample I	11.7		
Sample II	6.2		
Sample III	29.4		
Sample IV	12.4		
Sample V	19.1		
NOEL/1,000, 0.1735 mg/Kg/day			
Sample I	28.3		
Sample II	29.8		
Sample III	6.9		
Sample IV	19.3		
Sample V	24		
NOEL/200, 8.675 mg/Kg/day			
Sample I	22.1		
Sample II	8.7		
Sample III	19.1		
Sample IV	16.4		
Sample V	29.3		

Table S2. Literature for animal studies on triclosan, diethyl phthalate and methyl paraben.

Chemicals	Source	Title	Animal	Dose	Administration route	Duration of exposure	Outcome
Triclosan	(Louis et al. 2013)	The effect of triclosan on the uterotrophic response to extended doses of ethinyl estradiol in the weanling rat	Weanling Wistar rats	The series of TCS concentrations included 2.3, 4.69, 9.375, 18.75, and 37.5 mg/kg BW	Oral gavage	Postnatal days: 19-21	To evaluate the effect of TCS co-exposure with lower doses of ethinyl estradiol
Triclosan	(Paul et al. 2012)	Developmental triclosan exposure decreases maternal, fetal, and early neonatal thyroxine: a dynamic and kinetic evaluation of a putative mode-of-action	Time-pregnant Long-Evans female rats	0, 10, 30, 100, and 300 mg/kg/day	Oral gavage	From gestational day 6 through postnatal day 21	To examine whether TCS decreases thyroline (T4) in dams and offspring via up-regulation of hepatic catabolism
Triclosan	(Jung et al. 2012)	Potential estrogenic activity of triclosan in the uterus of immature rats and rat pituitary GH3 cells	Sprague-Dawley females immature rats	TCS at doses of 7.5, 37.4, and 187.5 mg/kg	Oral gavage	Postnatal days: 19-21	To screen estrogenic activity of TCS in the uteri of immature rats
Triclosan	(Rodriguez and Sanchez 2010)	Maternal exposure to triclosan impairs thyroid homeostasis and female pubertal development in Wistar rat offspring	Nulliparous female Wistar rats	Triclosan at dose of 0, 1, 10, or 50 mg/kg/d	Drinking water	8 days prior to mating; through gestation and lactation	To examine Effects of maternal exposure to triclosan on thyroid homeostasis (TH) and reproductive-tract development
Triclosan	(Paul et al. 2010a)	Developmental triclosan exposure decreases maternal and neonatal thyroxine in rats	Time-pregnant Long-Evans female rats	The dosing solutions (0, 30, 100, and 300 mg/ml)	Oral gavage	From gestational day 6 through postnatal day 21	To test the hypothesis that perinatal triclosan exposure will alter circulating thyroid hormone levels in pups during early postnatal development and in dams at the conclusion of lactation

Chemicals	Source	Title	Animal	Dose	Administration route	Duration of exposure	Outcome
Triclosan	(Stoker et al. 2010)	Triclosan exposure modulates estrogen-dependent responses in the female wistar rat	Female Wistar rat	Doses of triclosan: 1.18, 2.35, 4.69, 9.37, 18.75, 37.5, 75, 150, and 300 mg/kg	Oral gavage	Postnatal days: 22-42	To evaluate the effects of triclosan
Triclosan	(Wu et al. 2009)	Investigation on metabolism and pharmacokinetics of triclosan in rat plasma by using UPLC-triple quadrupole MS	Sprague-Dawley rats	Oral administration of 5 mg/kg triclosan	Oral administration	Single bolus	To understand the pharmacokinetics and metabolism of triclosan in animal and human body
Triclosan	(Paul et al. 2010b)	Short-term exposure to triclosan decreases thyroxine in vivo via upregulation of hepatic catabolism in Young Long-Evans rats	Female Long-Evans rats	The dosing solutions (0, 10, 30, 100, 300, and 1000 mg/kg/day)	Oral gavage	4 days	To test the hypothesis that triclosan decreases circulating T4 via upregulation of hepatic catabolism and transport
Triclosan	(Kumar et al. 2009)	Alteration of testicular teroidogenesis and histopathology of reproductive system in male rats treated with triclosan	Male Wistar rats, Rattus norvegicus	Three dose levels: 5, 10 and 20 mg/kg/day	Intubation	60 days	To elucidate the probable mode of action of TCS as an antiandrogenic compound
Triclosan	(Zorrilla et al. 2009)	The effects of triclosan on puberty and thyroid hormones in male Wistar rats	Weanling rats	0, 3, 30, 100, 200, or 300 mg/kg of triclosan	Oral gavage	Postnatal days: 23-53	To determine effects of triclosan on pubertal development and thyroid hormone concentrations
Triclosan	(Crofton et al. 2007)	Short-term in vivo exposure to the water contaminant triclosan: Evidence for disruption of thyroxine	Weanling female Long-Evans rats	The dosing solutions at concentrations of 0, 10, 30, 100, 300 and 1000 mg/kg/day	Oral gavage	Postnatal days: 27-29	To test the hypothesis that triclosan alters circulating concentrations of thyroxine

Chemicals	Source	Title	Animal	Dose	Administration route	Duration of exposure	Outcome
Diethyl phthalate	(Kwack et al. 2009)	Comparative toxicological evaluation of phthalate diesters and metabolites in Sprague-Dawley male rats for risk assessment	Sprague-Dawley male rats	The phthalate diesters (500 mg/kg/d)	Oral gavage	4 weeks	To comparatively assess the systemic toxicity and sperm parameters, nine phthalate diesters
Diethyl phthalate	(Pereira et al. 2007a)	Chronic toxicity of diethyl phthalate-A three generation lactational and gestational exposure study on male Wistar rats	Wistar rats	50mg/kg of the diet/day; 25mg/kg of the diet/day for F1 generation and 10mg/kg of the diet/day for F2 generation. 10, 25 and 50 mg/kg of the diet/day, which is equal to 0.57, 1.425 and 2.85 mg/kg body wt/day	Oral gavage	Throughout mating, gestation until termination at weaning	To understand the dose-response toxic effect of DEP over three generations in male Wistar rats
Diethyl phthalate	(Pereira et al. 2007b)	A two-generation chronic mixture toxicity study of Clophen A60 and diethyl phthalate on histology of adrenal cortex and thyroid of rats	Wistar rats	50mg/kg of the diet/day	Oral gavage	Throughout mating, gestation until termination at weaning	To observe the type of interaction that exists between polychlorinated biphenyls (Clophen A60) and diethyl phthalate (DEP) on the adrenal and thyroid glands
Diethyl phthalate	(Pereira and Rao 2006)	Combined and individual administration of diethyl phthalate and polychlorinated biphenyls and its toxicity in female Wistar rats	Female Wistar rats	with the diet at 50 mg/(kg diet day) (approximately 2.85 mg/(kg body weight day))	Oral gavage	150 days	To evaluate the interactive toxicity of DEP and PCBs in young female Wistar rats

Chemicals	Source	Title	Animal	Dose	Administration route	Duration of exposure	Outcome
Diethyl phthalate	(Pereira and Rao 2007)	Toxicity study of maternal transfer of polychlorinated biphenyls and diethyl phthalate to 21-day-old male and female weanling pups of Wistar rats	Wistar rats	50 mg/kg of the diet (2.85 mg/kg body wt)	Oral gavage	Throughout mating, gestation until termination at weaning	To evaluate the interactive toxicity of DEP and PCB in 21-day-old male and female pups of Wistar rats
Diethyl phthalate	(Fujii et al. 2005)	A two-generation reproductive toxicity study of diethyl phthalate (DEP) in rats	Crj:CD (SD) IGS rats	dietary dose levels of 0, 600, 3000 and 15000 ppm	Dietary exposure	4 weeks prior mating and throughout the subsequent breeding period until weaning of F1 pups at 3 weeks of age	To evaluate the effects of diethyl phthalate on parental reproductive performance
Diethyl phthalate	(Yamasaki et al. 2005)	Two-generation reproductive toxicity studies in rats with extra parameters for detecting endocrine disrupting activity: introductory overview of results for nine chemicals	Sprague-Dawley rats	at concentrations of 0, 600, 3000 or 15000 ppm in the diet	Dietary exposure	10 weeks prior to mating; for females, through gestation and lactation; and through autopsy for both sexes	To investigate the endocrine-mediated effects of nine chemicals including , diethyl phthalate
Diethyl phthalate	(Shiraishi et al. 2006)	Subacute oral toxicity study of diethylphthalate based on the draft protocol for "Enhanced OECD Test Guideline no. 407"	Sprague-Dawley rats	Doses of 0, 40, 200, and 1,000 mg/kg/day	Oral gavage	28 days	To investigate whether DEP has endocrine-mediated properties
Diethyl phthalate	(Sonde et al. 2000)	Simultaneous administration of diethylphthalate and ethyl alcohol and its toxicity in male Sprague-Dawley rats	Sprague-Dawley rats	50 ppm DEP (w/v)	Drinking water	120 days	To evaluate the interactive toxicity of DEP with ethyl alcohol (EtOH) in young male Sprague-Dawley rats

Chemicals	Source	Title	Animal	Dose	Administration route	Duration of exposure	Outcome
Diethyl phthalate	(Field et al. 1993)	Developmental toxicity evaluation of diethyl and dimethyl phthalate in rats	Crl:CD (SD)BR VAF/Plus outbred Sprague-Dawley rats (CD rats)	Doses of 0, 0.25, 2.5, and 5.0% DEP in feed were administered which provided daily doses of approximately 0, 0.20, 1.91, and 3.21 g DEP/kg	Dietary exposure	Gestational days: 6-15	Timed-pregnant rats were administered DEP or DMP in feed during organogenesis, and evaluations were made of maternal toxicity and effects on embryoifetal viability, growth and morphogenesis
Methyl paraben	(Vo et al. 2010)	Potential estrogenic effect(s) of parabens at the prepubertal stage of a postnatal female rat model	Sprague-Dawley rats	62.5, 250 or 1000 mg/kg BW/day	Dietary exposure	Postnatal days: 21-40	To examine the effects of parabens
Methyl paraben	(Hoberman et al. 2008)	Lack of effect of butylparaben and methylparaben on the reproductive system in male rats	Male Wistar rats	Diets containing 0, 100, 1000 or 10,000 ppm	Dietary exposure	8 weeks	To evaluate potential reproductive effects
Methyl paraben	(Oishi 2004)	Lack of spermatotoxic effects of methyl and ethyl esters of p-hydroxybenzoic acid in rats	Crj:Wistar rats	Doses of 0.1% and 1.0% each in the rat's diet	Dietary exposure	8 weeks	it is demonstrated that the methyl and ethyl esters of p-hydroxybenzoic acid do not have an adverse effect on male reproductive functions in rats

References

Crofton KM, Paul KB, Devito MJ, Hedge JM. 2007. Short–term in vivo exposure to the water contaminant triclosan: Evidence for disruption of thyroxine. Environ Toxicol Pharmacol 24:194–197.

Field EA, Price CJ, Sleet RB, George JD, Marr MC, Myers CB, et al. 1993. Developmental toxicity evaluation of diethyl and dimethyl phthalate in rats. Teratology 48:33–44.

Fujii S, Yabe K, Furukawa M, Hirata M, Kiguchi M, Ikka T. 2005. A two–generation reproductive toxicity study of diethyl phthalate (DEP) in rats. J Toxicol Sci 30 Spec No.:97–116.

Hoberman AM, Schreur DK, Leazer T, Daston GP, Carthew P, Re T, et al. 2008. Lack of effect of butylparaben and methylparaben on the reproductive system in male rats. Birth Defects Res B Dev Reprod Toxicol 83:123–133.

Jung EM, An BS, Choi KC, Jeung EB. 2012. Potential estrogenic activity of triclosan in the uterus of immature rats and rat pituitary GH3 cells. Toxicol Lett 208:142–148.

Kumar V, Chakraborty A, Kural MR, Roy P. 2009. Alteration of testicular steroidogenesis and histopathology of reproductive system in male rats treated with triclosan. Reprod Toxicol 27:177–185.

Kwack SJ, Kim KB, Kim HS, Lee BM. 2009. Comparative toxicological evaluation of phthalate diesters and metabolites in Sprague–Dawley male rats for risk assessment. J Toxicol Environ Health A 72:1446–1454.

Louis GW, Hallinger DR, Stoker TE. 2013. The effect of triclosan on the uterotrophic response to extended doses of ethinyl estradiol in the weanling rat. Reprod Toxicol 36:71–77.

Oishi S. 2004. Lack of spermatotoxic effects of methyl and ethyl esters of p–hydroxybenzoic acid in rats. Food Chem Toxicol 42:1845–1849.

Paul KB, Hedge JM, Bansal R, Zoeller RT, Peter R, Devito MJ, et al. 2012. Developmental triclosan exposure decreases maternal, fetal, and early neonatal thyroxine: a dynamic and kinetic evaluation of a putative mode–of–action. Toxicology 300:31–45.

Paul KB, Hedge JM, Devito MJ, Crofton KM. 2010a. Developmental triclosan exposure decreases maternal and neonatal thyroxine in rats. Environ Toxicol Chem 29:2840–2844.

Paul KB, Hedge JM, Devito MJ, Crofton KM. 2010b. Short–term exposure to triclosan decreases thyroxine in vivo via upregulation of hepatic catabolism in Young Long–Evans rats. Toxicol Sci 113:367–379.

Pereira C, Mapuskar K, Rao CV. 2007a. Chronic toxicity of diethyl phthalate–A three generation lactational and gestational exposure study on male Wistar rats. Environ Toxicol Pharmacol 23:319–327.

Pereira C, Mapuskar K, Vaman RC. 2007b. A two–generation chronic mixture toxicity study of Clophen A60 and diethyl phthalate on histology of adrenal cortex and thyroid of rats. Acta Histochem 109:29–36.

Pereira C, Rao CV. 2006. Combined and individual administration of diethyl phthalate and polychlorinated biphenyls and its toxicity in female Wistar rats. Environ Toxicol Pharmacol 21:93–102.

Pereira C, Rao CV. 2007. Toxicity study of maternal transfer of polychlorinated biphenyls and diethyl phthalate to 21–day–old male and female weanling pups of Wistar rats. Ecotoxicol Environ Saf 68:118–125.

Rodriguez PE, Sanchez MS. 2010. Maternal exposure to triclosan impairs thyroid homeostasis and female pubertal development in Wistar rat offspring. J Toxicol Environ Health A 73:1678–1688.

Shiraishi K, Miyata K, Houshuyama S, Imatanaka N, Umano T, Minobe Y, et al. 2006. Subacute oral toxicity study of diethylphthalate based on the draft protocol for "Enhanced OECD Test Guideline no. 407". Arch Toxicol 80:10–16.

Sonde V, D'souza A, Tarapore R, Pereira L, Khare MP, Sinkar P, et al. 2000. Simultaneous administration of diethylphthalate and ethyl alcohol and its toxicity in male Sprague–Dawley rats. Toxicology 147:23–31.

Stoker TE, Gibson EK, Zorrilla LM. 2010. Triclosan exposure modulates estrogen–dependent responses in the female wistar rat. Toxicol Sci 117:45–53.

Vo TT, Yoo YM, Choi KC, Jeung EB. 2010. Potential estrogenic effect(s) of parabens at the prepubertal stage of a postnatal female rat model. Reprod Toxicol 29:306–316.

Wu J, Yue H, Cai Z. 2009. Investigation on metabolism and pharmacokinetics of triclosan in rat plasma by using UPLC–triple quadrupole MS. Se Pu 27:724–730.

Yamasaki K, Takahashi M, Yasuda M. 2005. Two–generation reproductive toxicity studies in rats with extra parameters for detecting endocrine disrupting activity: introductory overview of results for nine chemicals. J Toxicol Sci 30 Spec No.:1–4.

Zorrilla LM, Gibson EK, Jeffay SC, Crofton KM, Setzer WR, Cooper RL, et al. 2009. The effects of triclosan on puberty and thyroid hormones in male Wistar rats. Toxicol Sci 107:56–64.
